# Frequency of obesity and comorbidities in medical students

**DOI:** 10.12669/pjms.326.10492

**Published:** 2016

**Authors:** Yasir Mehmood, Farhan Khashim Al-Swailmi, Shehab Ahmed Al-Enazi

**Affiliations:** 1 Yasir Mehmood, FCPS. Assistant Professor of Surgery, Department of Surgery, Faculty of Medicine, Northern Border University Arar, Kingdom of Saudi Arabia; 2 Farhan Khashim Al-Swailmi, MD. Assistant Professor, Department of Ophthalmology, Faculty of Medicine, Northern Border University Arar, Kingdom of Saudi Arabia; 3 Shehab Ahmed Al-Enazi, MD. Assistant Professor, Department of Pediatrics, Faculty of Medicine, Northern Border University Arar, Kingdom of Saudi Arabia

**Keywords:** Obesity, Frequency, Medical students

## Abstract

**Objectives:**

To determine the frequency of obesity disorders and their co-morbidities in medical students.

**Methods:**

This cross-sectional study was conducted in Faculty of Medicine, Northern Border University, Ar’ar, Saudi Arabia. All medical students who consented to participate were included in the study. Their relevant information was recorded on a structured proforma. Weight and height of the participants were measured using calibrated manual weighing scale and Body mass index (BMI) was calculated. The obtained results were interpreted according to classification of body weight disorders. The participants who turned out to be over-weight and obese were further assessed for hypertension, diabetes mellitus and gallstones. The collected data was analyzed using the Statistical Package for Social Science (SPSS) version 20.

**Results:**

A total of 405 students participated in study, age range was 19-25 years. Male were 169 (41.7%) and female students were 236(58.3%). Family history of obesity was present in 34.3%. Out of 405 students, 126 were having BMI between 25 and 45.6, among them 34(8.4%) students were obese and 88 (21.7%) were overweight. Sixty two (15.3%) among them were male and 64 (15.8%) female. Fourteen (11.1%) were hypertensive and 9(7.1%) were having gall stones.

**Conclusion:**

The frequency of obesity among medical students was 8.4%. Increasing frequency of obesity associated with unhealthy life style needs to be controlled at national level to raise a healthy generation and to reduce burden on health economy.

## INTRODUCTION

Obesity is a public health and policy problem because of its prevalence, costs, and health effects. A person is considered overweight if her/his body mass index (BMI) is 25-29.9, and obese if BMI is over 30.^1^

The incidence of obesity has increased rapidly during recent decades. More than 30% of Americans are obese, as are more than a quarter of men and women in several European countries.^2^ Obesity is not just a cosmetic consideration. The metabolic changes of obesity can induce serious health problems and can increase the risk of many diseases like hypertension, dyslipidaemia, diabetes mellitus, orthopedic complications, gallstones, breast cancer and psychological disorders.^3^,^4^ The number of years that one lives with obesity, is directly associated with the risk of mortality.^5^ According to the World Health Organization (WHO), the world-wide obesity has more than doubled since 1980, and in 2008.^6^

Obesity is increasing in the Kingdom of Saudi Arabia at an alarming rate. Based on the National Nutrition Survey of 2007 the prevalence of obesity in the KSA was 23.6% in women and 14% in men. The prevalence of overweight in the community was determined to be 30.7% for men as compared to 28.4% for the women.^7^ A study on college students in Rass showed 21.8% of the students were overweight and 15.7% were obese.^8^

Today’s medical students are tomorrow’s doctors and they are considered in the community as the most knowledgable and health conscious population. There are very few studies conducted on medical students regarding their obesity in Saudi Arabia. Therefore this study was planned to determine frequency of obesity and its effects on their life in term of any co morbidities.

## METHODS

This cross-sectional study was conducted in Faculty of Medicine, Northern Border University, Arar, Saudi Arabia from September, 2015 to February, 2016. All male and female medical students who consented to participate were included in the study. Their relevant information was recorded on a structured proforma.

Weight and height of the participants were measured using calibrated manual weighing scale. Body mass index (BMI) was calculated by using the:

Quettlet’s formula: BMI = Weight (Kg)/Height (m)^2^

The obtained results were interpreted according to classification of body weight disorders. Student were labeled underweight if BMI less than 18.5, normal if BMI between 18.6 and 24.9, overweight if BMI 25 and 29.9 and obese if BMI turned out 30 or more. The participants who turned out to be over-weight and obese were further assessed for hypertension by blood pressure monitoring, diabetes mellitus and gallstones with the help of ultrasound. Blood pressure exceeding 140 over 90 mmHg on two consecutive days was considered hypertension. High blood sugar was defined as fasting blood sugar level > 126 mg/dl on two consecutive days. The collected data was analyzed using the Statistical Package for Social Science (SPSS) version 20. Permission to conduct this research was sought from Institution Review Board of the Faculty of Medicine with reference No. 481/2015.

## RESULTS

**Fig.1:**
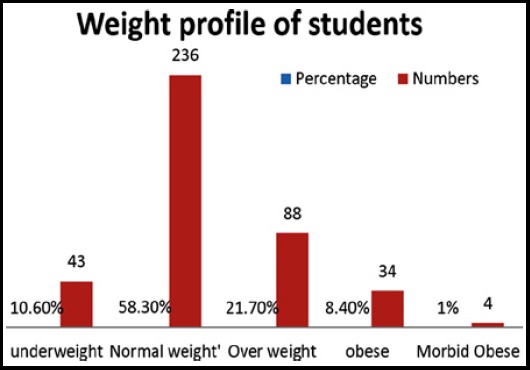
Weight profile of all students.

A total 405 students participated in study. Mean age was 21.49 ±1.59 with age range of 19-25 years. Male were 169 (41.7%) and female students were 236(58.3%). Family history of obesity was present in 139 (34.3%). Out of 405 students, 34(8.4%) students were obese. The weight profile of other students is given in Fig.1.

Out of 405 students, 126 were having BMI between 25 and 45.6. Among them male were 62 (15.3%) were and female were 64 (15.8%). Out of these 126 students, 14 (11.1%) were hypertensive and 9(7.1%) were having ultrasound evidence of gall stones. Weight disorders and hypertension has shown significant positive correlation with each other when analyzed in total study subjects. (Table-I)

**Table-I:** Weight disorder * Hypertensive Cross tabulation.

			Hypertensive		Total	Pearson chi square	P value

			Yes	No			
Weight disorder	Over weight	Count	4	84	88	23.46	0.000
		% within Weight disorder	4.5%	95.5%	100.0%		
		% within Hypertensive	28.6%	75.0%	69.8%		
	Obese	Count	7	27	34		
		% within Weight disorder	20.6%	79.4%	100.0%		
		% within Hypertensive	50.0%	24.1%	27.0%		
	Morbid obesity	Count	3	1	4		
		% within Weight disorder	75.0%	25.0%	100.0%		
		% within Hypertensive	21.4%	0.9%	3.2%		
Total		Count	14	112	126		
		% within Weight disorder	11.1%	88.9%	100.0%		
		% within Hypertensive	100.0%	100.0%	100.0%		

Out of 126 students with obesity disorders, 74(58.7%) were having family history of obesity while in 52(41.3%) there was no family history. Junk food eating for more than twice a week was present in 92(73%) while 34(27%) were suffering from obesity even without eating junk foods. Out of 126, Ninety six (76.2%) were not involved in sports activity while 30(23.8%) were doing some sports activities.

## DISCUSSION

The obesity epidemic has taken the Middle East in its grip, bringing along a tide of chronic disease that will severely affect population’s well-being and test national health systems. The problem of obesity is growing in Kuwait, Oman, Saudi Arabia, Lebanon, Turkey, Bahrain and Jordan. Obesity is on top in Kuwait and it will reach exceedingly high levels by 2030. Saudi Arabian men and women also have consistently high levels of overweight.^9^ The region of Middle East and North Africa had the seventh highest prevalence of obesity in men, and the second highest in women between 1980 and 2008.^10^

The economic growth and prosperity of Saudi Arabia have brought pronounced changes in the lifestyle of the people. Over the last two decades the influence of the western world has led to an increased consumption of fast foods and sugar-dense beverages. Simultaneously, technological advances – cars, elevators, escalators and remotes have lead to a decrease in level of physical activity. Consequently, obesity is increasing in the Kingdom at an alarming rate.^11^ Sudip Paul^12^ conducted an obesity study on university students of Bangladesh. He has reported 25% overweight that is almost similar to our findings (21.7%) but rate of obesity (4%) in his study is significantly less our findings (8.4%). Gross difference in obesity may be attributed to the difference in economic prosperity of two countries.

Zaytoun S^13^ has found that 6.2% of the university students were obese in a study sample of 500. Interestingly, his sample size and results are closer to our study sample and results. Contrary to Zaytoun S, Bakr^14^ has reported 12.5% obesity and Farahat and Abou-El Fath^15^ found that 13.4% of the university students were obese in same country- Egypt.

Mukhtiar Baig^16^ has found 19% of the students were obese in the study conducted on university students in Jeddah, Saudi Arabia. Coronary Artery Disease in Saudis Study (CADISS) of 2005 estimated an overall obesity prevalence of 35.5% in the Kingdom: in other words one in every three people in the country is obese.^17^ Prevalence of overweight and obesity in adult Kuwaiti population were 80.4% and 47.5%, respectively. Overweight and obesity rates were higher in women 81.9% and 53% compared to men 78% and 39.2%.^18^,^19^

The prevalence of obesity was examined among 222 Saudi female medical and nursing students by Rasheed P, Abou-Hozaifa BM and Khan A. Rate of obesity in their study was 30.6%. The prevalence of obesity in these young Saudi women was notably high and supports findings of earlier studies for a common occurrence of female obesity in this region.^20^ Similarly in another study on 894 Saudi male adolescents (age 12-20 years), the prevalence of overweight was 13.8% and obesity was 20.5%. Family history and lack of physical activity were associated with adolescent obesity.^21^

In another study on primary school children in Saudi Arabia, the prevalence of overweight among children was 14.2% while obesity was 9.7%. These all cases were associated with positive family history of obesity.^22^ The prevalence of overweight and obesity were 20% and 11%, respectively among female school-aged children and adolescents in primary and intermediate schools of Saudi Arabia. The students’ ages ranged from 6 to 17 years.^23^ A study conducted by Ibrahim et al. on medical students in Jeddah University revealed that 19.1% were overweight and 12.7% were obese. In our study 21.7% were overweight and 8.4% were obese.^24^

There is a direct association between obesity and several diseases like diabetes mellitus, hypertension, dyslipidaemia and ischemic heart disease.^25^ Weight gain appears to precede the development of diabetes.^26^ The Swedish Obesity Study showed hypertension to be present at baseline in 44-51% of obese subjects.^27^ Excess body weight may account for up to 26% of cases of hypertension in men and 28% in women.^28^ Hypertension rate was 11.1% in our study while 11.2% of the university students were suffering from hypertension in Sudip Paul’s study.^12^ Weight loss in obese subjects is associated with a decline in blood pressure. A 10% weight loss was independently associated with a 4.3/3.8 mmHg decrease in 24-h ambulatory blood pressure monitoring in 4-year follow up of 181 overweight hypertensive patients.^29^

Women with BMI> 45kg/m2 had a seven fold increase in risk for gallstones compared to women with BMI<24kg/m2. Women with BMI>30kg/m2 had a yearly gallstone incidence of >1%.^30^ Gall stones were present in 7.1% in our study.

Further research is needed to clarify association between current economic policies of Saudi Arabia and existing risk factors for obesity to help policy makers to decide on future action plans.

## CONCLUSION

The prevalence of overweight and obesity among medical students was 21.7% and 8.4% respectively in our study. Although our results are relatively better due to awareness in medical students regarding morbidity and mortality associated with obesity but obesity is increasing among those who are using unhealthy life style, including fast food and fried snacks consumption. This study reinforces the need to encourage healthy lifestyle and healthy food habits. Risk of co-morbidities increases with increasing obesity. Our results are important for policy makers to know the effectiveness of obesity interventions on future disease burden because government has to spend lot of resources to build up specialized centers to manage co morbidities associated with diabetes.
